# Supplementary material for: Comparative Gravimetric Studies on Carbon Steel Corrosion in Selected Fruit Juices and Acidic Chloride Media (HCl) at Different pH
Source: Materials (Basel). 2021 Aug 23;14(16):4755. doi: 10.3390/ma14164755 (PMC8400660; doi:10.3390/ma14164755)
Supplement: Supplementary file 1 [file materials-14-04755-s001.zip › materials-1314450-supplementary.pdf]

materials

MDPI

Supplementary Materials

Comparative Gravimetric Studies on Carbon Steel Corrosion in Selected Fruit Juices Processing Environments and Acidic Chloride Media (HCl) at different pH.

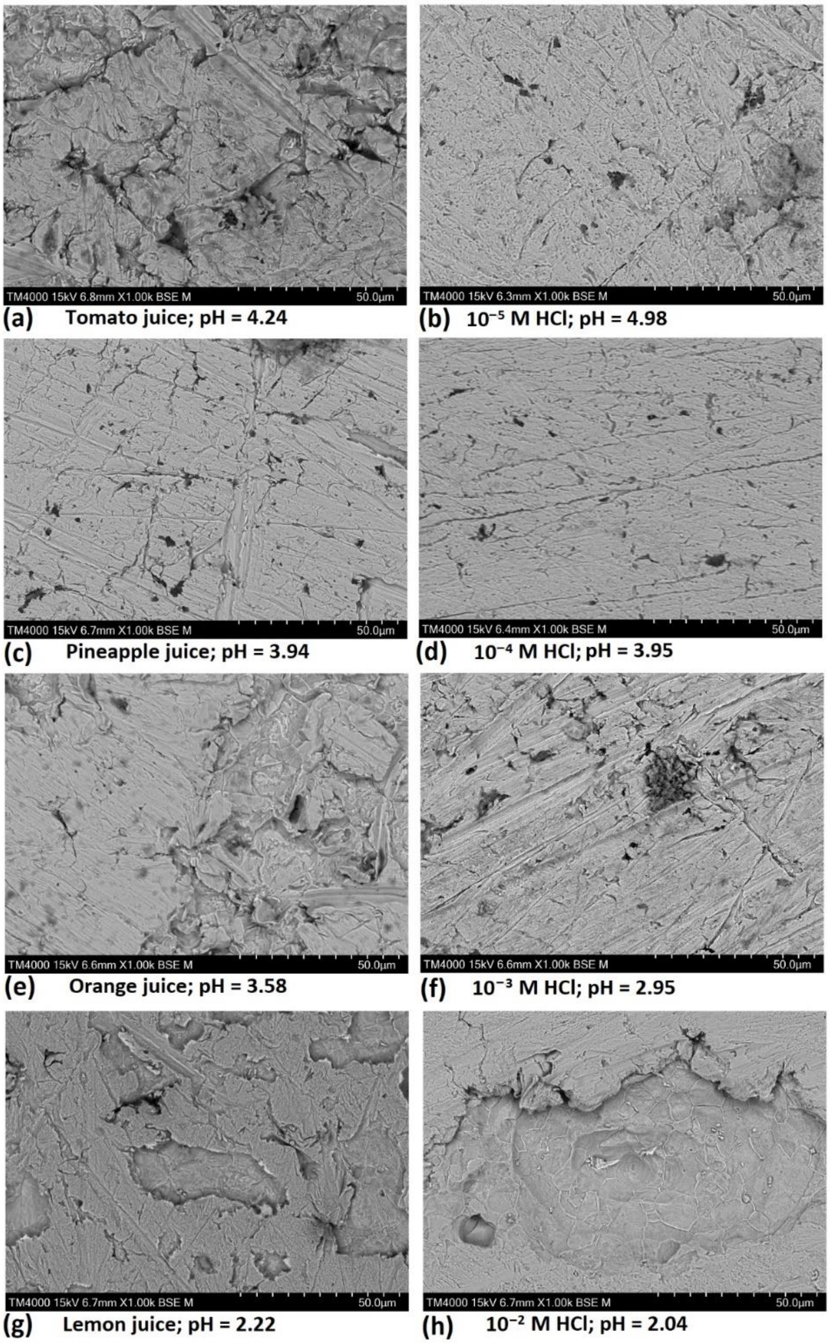

Figure S1. SEM images of carbon steel sheet (CR1 ≈ EN 1.0338) surfaces acquired at 15 kV after immersion in (a) tomato juice, (b) 10-5 M HCl, (c) pineapple juice, (d) 10-4 M HCl, (e) orange juice,, (f) 10-3 M HCl,, (g) lemon juice, and (h) 10-2 M HCl, respectively.

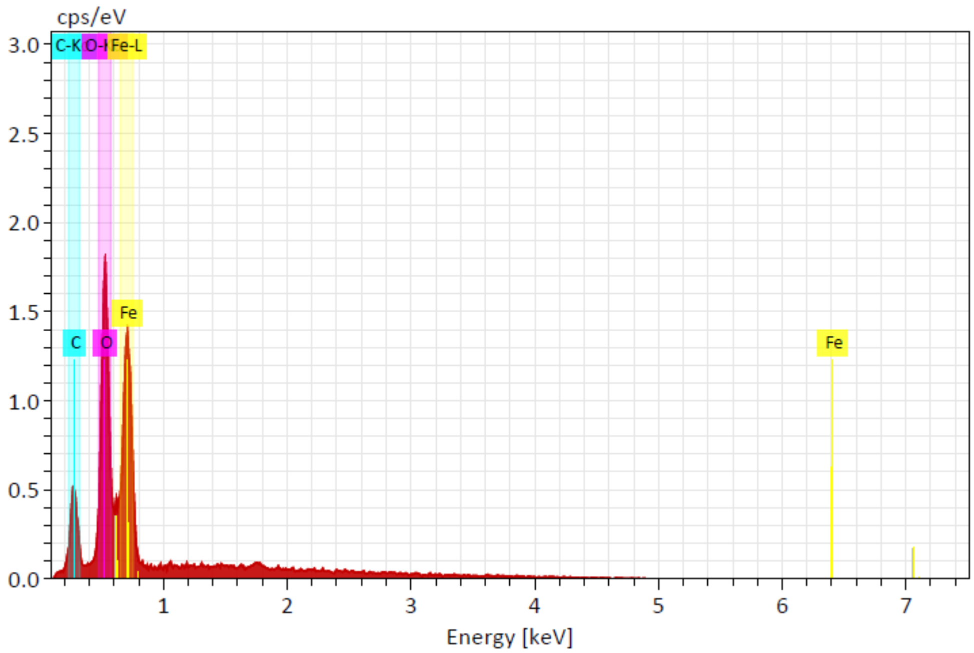

Figure S2. EDX spectra acquired during mapping of carbon steel sheet exposed to tomato juice.

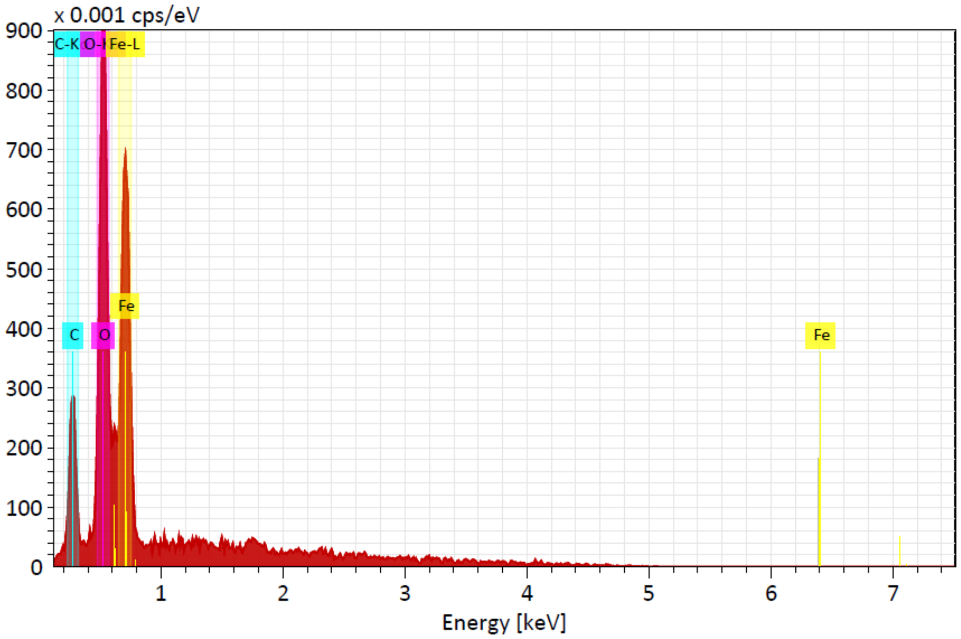

Figure S3. EDX spectra acquired during mapping of carbon steel sheet exposed to pineapple juice.

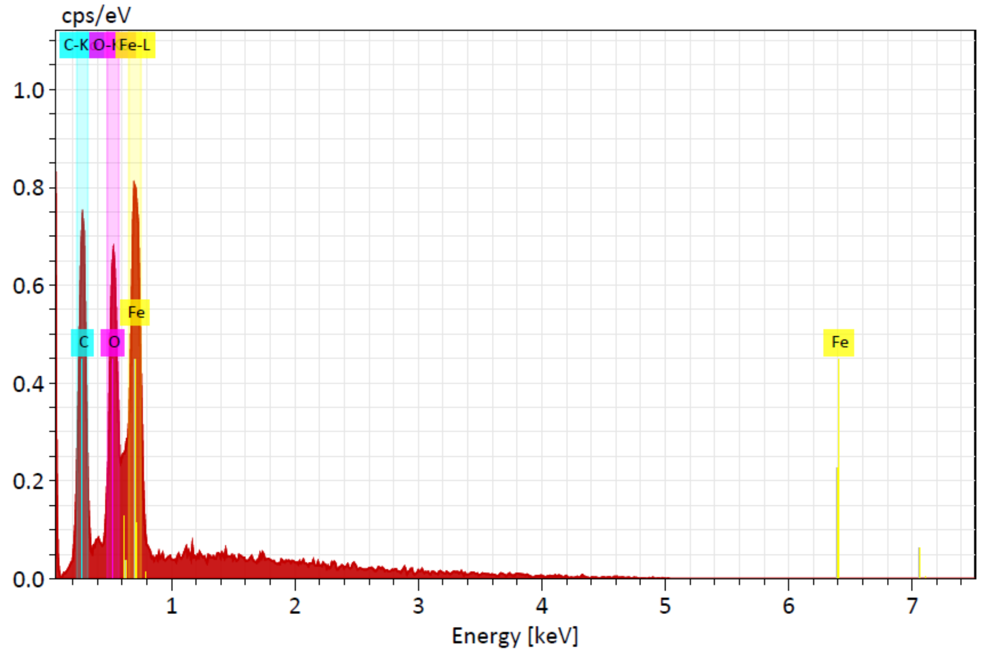

Figure S4. EDX spectra acquired during mapping of carbon steel sheet exposed to orange juice.

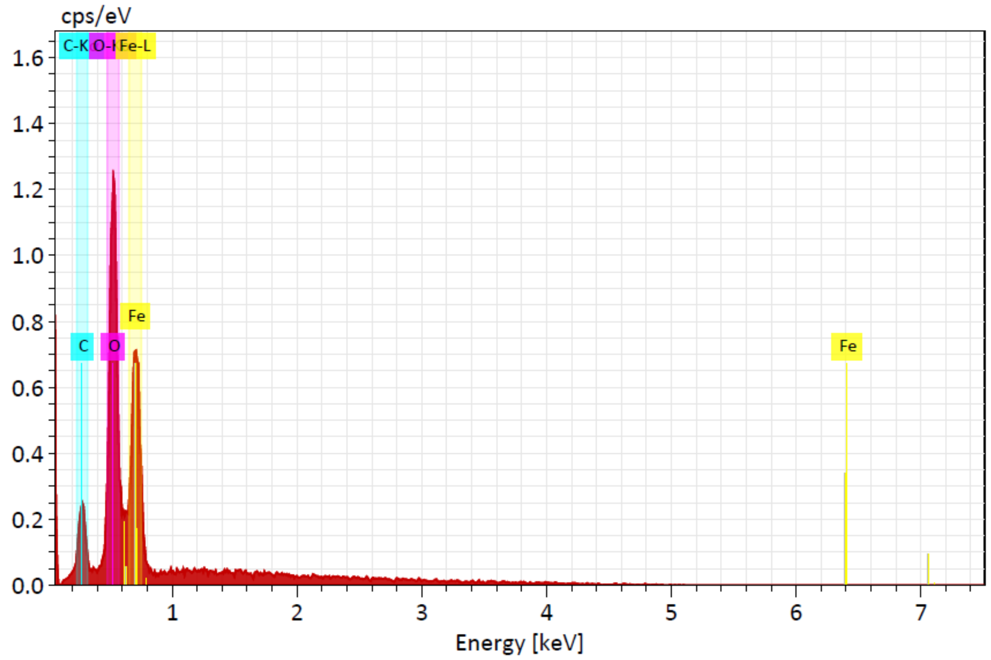

Figure S5. EDX spectra acquired during mapping of carbon steel sheet exposed to lemon juice.
